# Lupus enteritis masquerading as Crohn’s disease

**DOI:** 10.1186/s12876-019-1058-1

**Published:** 2019-08-27

**Authors:** Xiu-Li Zhu, Xue-Mei Xu, Si Chen, Qiao-Min Wang, Kai-Guang Zhang

**Affiliations:** Department of Gastroenterology, Anhui Provincial Hospital, Hefei, Anhui 230001 People’s Republic of China

**Keywords:** Systemic lupus erythematosus, Crohn’s disease, Lupus enteritis

## Abstract

**Background:**

Systemic lupus erythematosus is an autoimmune disease which can affect multiple organs, resulting in significant mortality and morbidity. Lupus enteritis is one of the rare complications of SLE, defined as vasculitis of the intestinal tract, with supportive biopsy findings and/or image. However, lupus enteritis is seldom confirmed on histology or image and the changes of intestinal mucosa are nonspecific. Crohn’s disease is a chronic inflammatory disorder of the gastrointestinal tract which affects any part of the gastrointestinal tract. The diagnosis of CD is confirmed by clinical evaluation and a combination of endoscopic, histology, radiology, and/or biochemical investigations.

**Case presentation:**

Here we report a rare case of a 71-years-old Chinese male has been diagnosed with lupus enteritis which similar to CD in the aspects of endoscopic, histology, and radiology. So far, there are no relevant cases reported.

**Conclusions:**

The endoscopic appearance of lupus enteritis is nonspecific, on the basis of our case, the features of lupus enteritis can be described as spacious, clean and no moss ulcers which discontinuous involved all gastrointestinal tract.

## Background

Systemic lupus erythematosus (SLE) is a multifactorial autoimmune disorder in which the body’s immune system mistakenly attacks healthy tissue. The disease is gender-related occurring nine times more likely in women than men, especially in women of child-bearing years (15 to 35 years old) [1]. SLE usually damages the heart, joints, skin, lungs, blood vessels, kidneys, and nervous system. In recent years, some studies have reported that SLE also harms gastrointestinal tract and lupus enteritis as an initial manifestation of SLE. There is no standard definition of lupus enteritis, most scholars believe that lupus enteritis is vasculitis or intestinal inflammation with supportive images and/or biopsy results. But it is so difficult to diagnose lupus enteritis relying on histology and radiology in the clinic. Crohn’s disease (CD) is a lifelong disease caused by the interaction between infectious, immune, genetic and environmental factors. A single gold standard for the diagnosis of CD is not available. The current view is that diagnosis is based on a combination of clinical manifestations, endoscopic appearance, radiology, histology, and surgical outcomes, however, this still results in diagnostic obstacles [2]. The two diseases are rare and the diagnosis is difficult. The following case describe an old male presenting with lupus enteritis and diarrhea as the initial manifestation of SLE, but in terms of endoscopy, histology, and radiology, the case is similar to CD. To the best of our knowledge, there are no relevant cases reported in the English literature.

## Case presentation

**Fig. 1:**
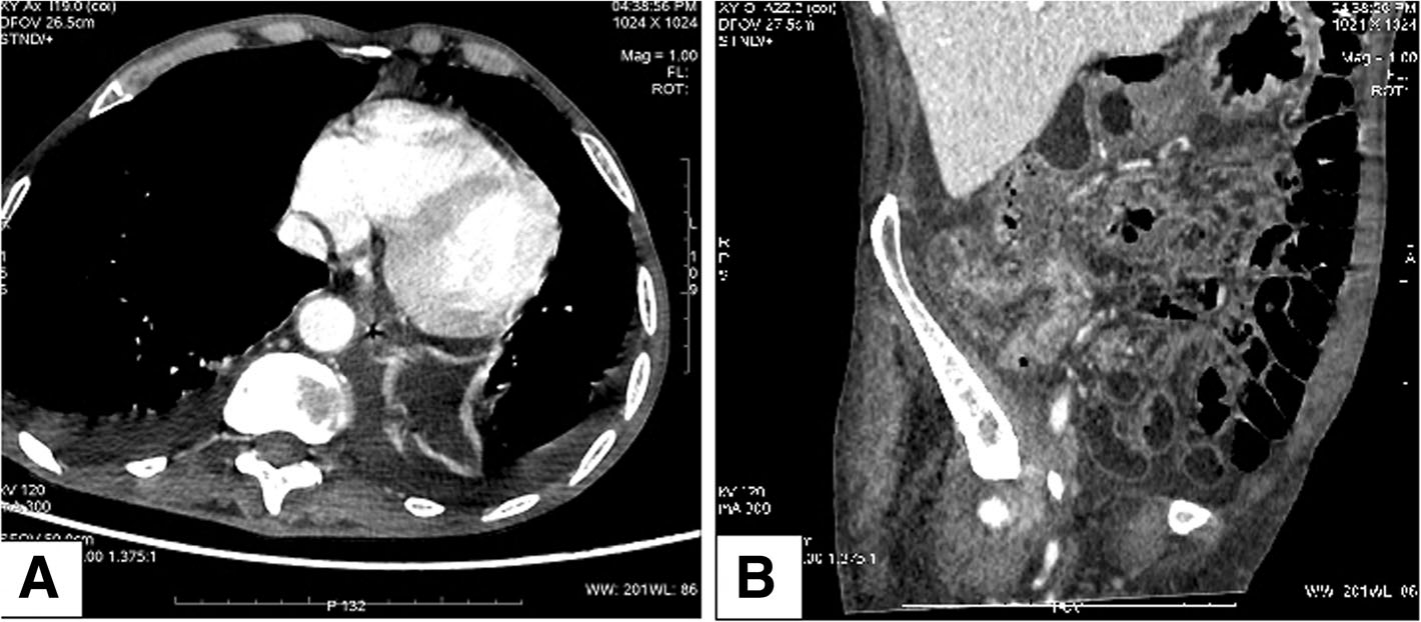
Chest and abdominal enchaned CT revealed polyserositis (pleural effusion, ascites, pericardial effusion) (**a**) and marked thickening of the entire colonic mucosa (**b**)

**Fig. 2:**
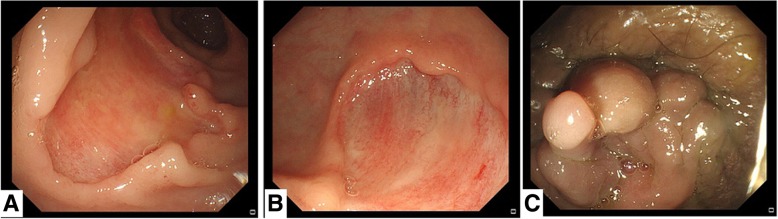
Electronic colonoscopy showed multiple ulcers in the terminal ileum and colon which were circular, wide, clean, without moss (**a**, **b**) and hyperplastic lesions around anus (**c**)

**Fig. 3:**
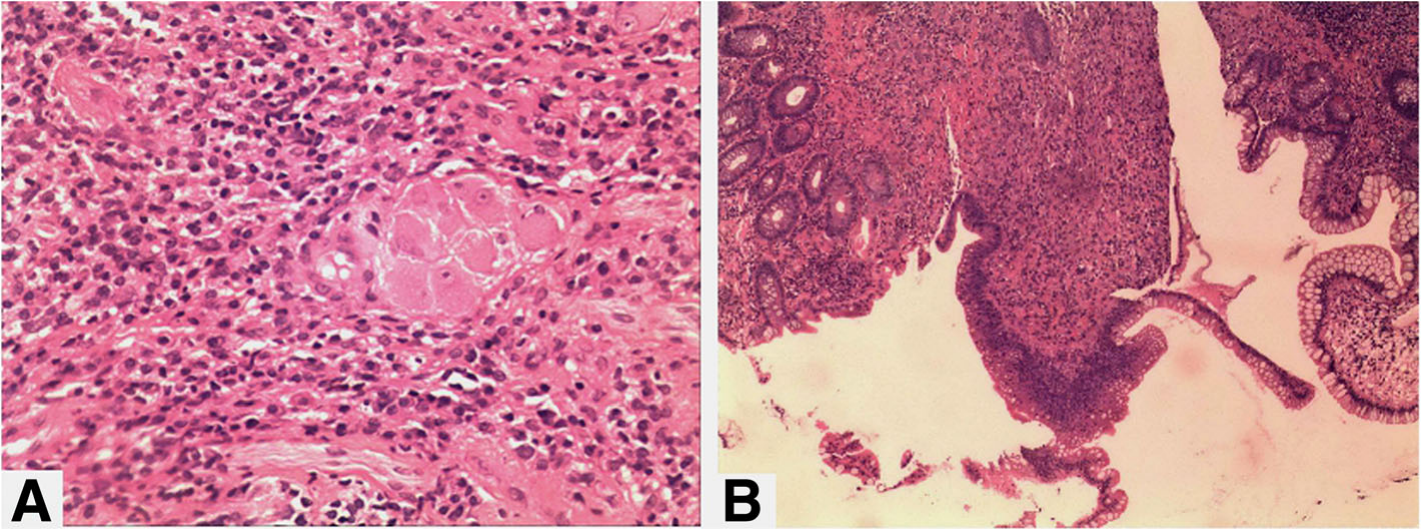
Intestinal histopathology showed ganglion cells, Inflammatory cell infiltration (**a**) and crack shape ulcer (**b**)

A 71-year-old Chinese male with no significant medical history was admitted to the department of gastroenterology in our hospital with three months of watery diarrhea and mild abdominal pain. The patient described the diarrhea frequency was six to ten times per day without mucoid or blood. Physical examination revealed one oral ulcer, tenderness of the abdomen without rebound tenderness and shifting dullness. Laboratory tests revealed a leukocyte count of 12.5*10^9^/L, anemia (hemoglobin of 67 g/L) and a positive antinuclear antibody titer of 1:3200, positive serology for the antiphospholipid antibody. Erythrocyte sedimentation rate (ESR) and C-reactive protein (CRP) were 130 mm/h and 117 mg/L, respectively (normal: 0-15 mm/h and 0–8 mg/l, respectively). Complement components C3 and C4 were 50 mg/dL and 12 mg/dL, respectively (normal: 86-160 mg/dL and 17-45 mg/dL, respectively). Syphilis serology and TPPA/TPHA, TRUST were positive. PPD experiment and T-spot test were negative. Fecal routine, fecal bacteriological tests (C.difficile, Salmonella, Campylobacter, Yersinia and many more) and fecal virological tests were all normal. Contrast-enhanced computed tomography (CT) of the chest and abdomen revealed polyserositis (pleural effusion, ascites, pericardial effusion) and marked thickening of the entire colonic mucosa (Fig. 1). Ascites routine revealed pale yellow and Rivalta test(+), quantitative counting of nucleated cells were 462*10^6^/L and monocytes (72%), coenocyte (27.7%). Electronic colonoscopy showed multiple ulcers in the terminal ileum and colon which were circular, wide, clean, without moss and hyperplastic lesions around the anus (Fig. 2). The pathology tended to CD because there were ganglion cell and crack shape ulcer (Fig. 3). Gastroscopy showed no obvious abnormalities in another hospital.

Through the case discussion in the multi-disciplinary team (MDT) including histology, radiology, rheumatology, and gastroenterology we diagnosed the patient with lupus enteritis prior to CD. Then the patient was given systemic steroids (Solu-Medrol 80 mg QD) and within a few days (7–10 days) the abdominal pain and diarrhea began to resolve. The patient was discharged on a tapering dose of steroids (Prednisone 50 mg QD). The CRP, ESR, Complement components C3 and C4 were normal after two months.

**Fig. 4:**
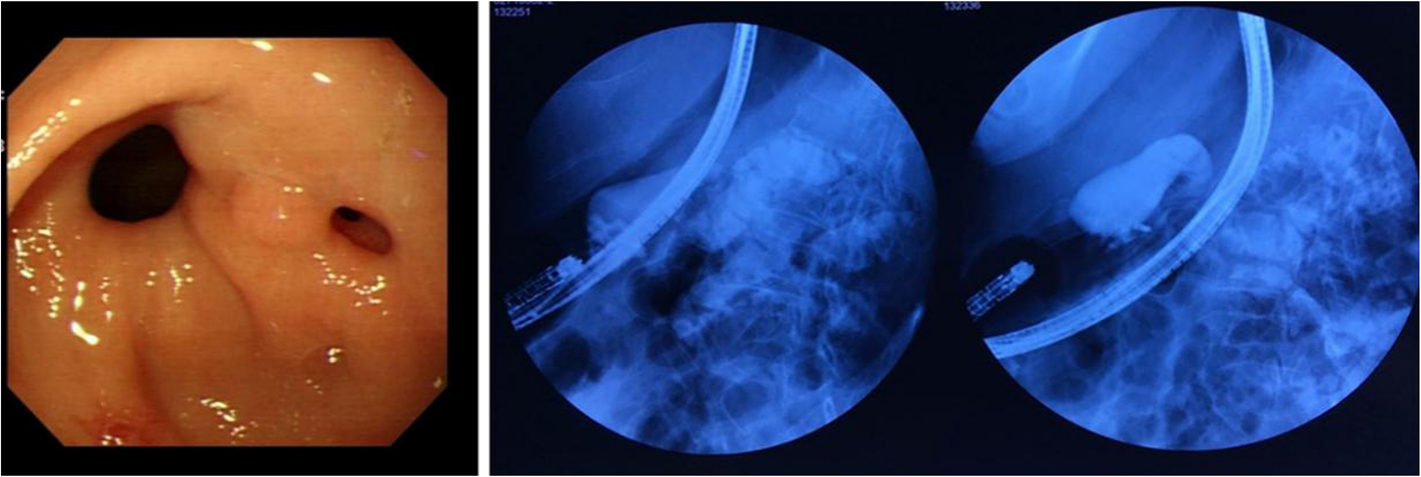
Gastroscopy combined with angiography showed there was gastric-intestinal fistula

**Fig. 5:**
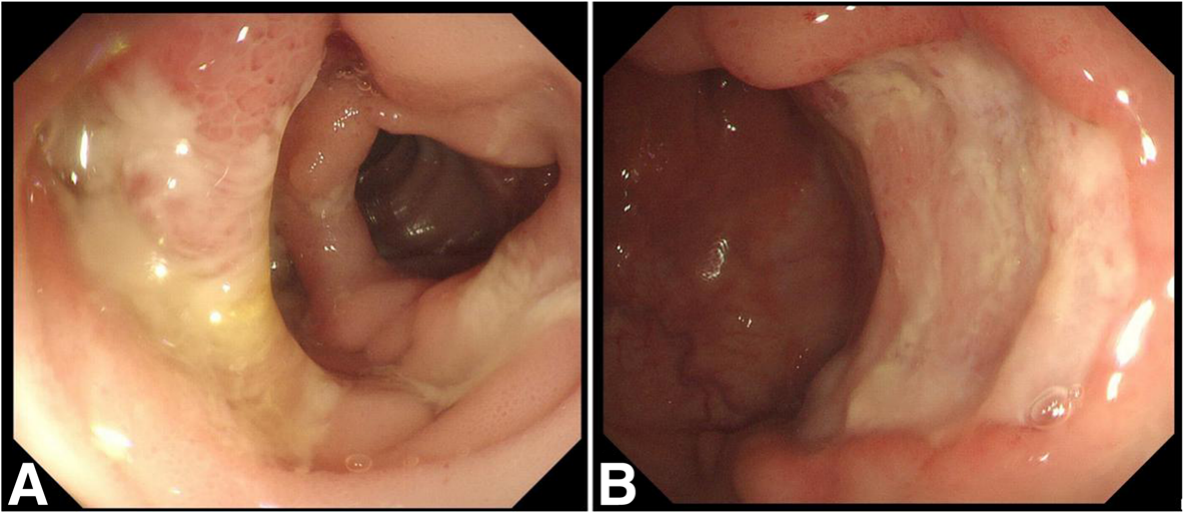
Electronic colonoscopy showed no significant improvement compared with the previous (**a**, **b**), intestinal ulcer surface covered with white moss (**a**)

However, the patient complained of bloating after a meal three months after discharge, gastroscopy combined with angiography showed there was gastric-intestinal fistula (Fig. 4). Electronic colonoscopy showed no significant improvement compared with the previous, intestinal ulcer surface covered with white moss (Fig. 5). The patient had severe abdominal pain soon, we considered there was digestive tract perforation and recommended surgery, his family members refused further treatment due to economic reasons and he died after one day.

## Discussion and conclusions

The American College of Rheumatology (ACR) established eleven criteria in 1982, which were revised in 1997 as a classification tool for implementing SLE definitions in clinical trials [3]. If a person has any 4 out of 11 symptoms, he is with SLE. In our case, the old male meets five criteria: (1) oral ulcer; (2) polyserositis including pleural effusion, ascites and, pericardial effusion; (3)anemia; (4) positive serology for antiphospholipid antibody and syphilis; (5) positive antinuclear antibody titer of 1:3200. From the above, the patient with SLE is clear. The multiple intestinal ulcers need differential diagnosis between CD and lupus enteritis. CD is a chronic and disabling inflammatory disease of the digestive system of unknown etiology. The incidence of CD is highest in the Northern Europe, more and more young people have been diagnosed CD in China recently [4]. The diagnosis of CD is established by a combination of clinical symptoms, endoscopic appearance, radiology and histology. Chronic diarrhea is the most common symptom in CD. The most significant endoscopic features of CD are discontinuous involvement, anal lesions, and cobble stoning. CT is an imaging technique with the highest diagnostic accuracy for the detection of intestinal involvement and penetrating lesions in CD, the features of CD are dilation, tortuosity, prominence and wide spacing of mesentery with mesenteric arterial which called comb’s sign [5]. Focal crypt irregularity and granulomas are the generally accepted pathological features. There are some aspects support CD: (1) chronic diarrhea; (2) marked thickening of the entire colonic mucosa; (3) pathological tend to CD. However, there are some opposed points: (1) old male and short disease course; (3) endoscopic appearance is not totally conformed to CD. There is no consistent definition of lupus enteritis. Some scholars believe that lupus enteritis is the basis of a series of processes, including intestinal vasculitis, mesenteric arteritis, abdominal serositis and lupus peritonitis [6]. Most people think that lupus enteritis is vasculitis or inflammation of the intestinal tract. The most frequent symptoms were abdominal pain and diarrhea. CT and pathology have become the gold standard for diagnosis [7]. Typical features include bowel dilation,bowel-wall thickening, abnormal bowel wall enhancement (target sign) [8], engorgement of mesenteric vessels with an increased number of visible vessels (comb’s sign). These are nonspecific because the above-described abnormalities can also be seen in patients with pancreatitis, peritonitis or CD. There is no unified view about the endoscopic appearance of lupus enteritis, only one Chinese professor described “deep ulcer” in a case report of lupus enteritis. In our case, the ulcers of the terminal ileum and colon are similar to CD and “deep ulcer” in a few points. On the basis of diagnosis in patients with SLE, the endoscopic appearance was more inclined to lupus enteritis and treatment effect also confirmed this point. Whether SLE can cause gastrointestinal perforation nor not, less reported at present. Oshimo Y [9] reported a case of the elderly man with SLE associated with paralytic ileus and fistula formation in 1999, our case is the first case of SLE with gastric-intestinal fistula.

The differentiation of CD from SLE gastrointestinal involvement may be difficult. In fact, cases with inflammatory bowel diseases like CD could show similar clinical signs and symptoms to SLE, and in some cases of CD might fulfill some of the classifications of criteria for SLE. With reference to the literature [10], we listed the following differences between lupus-like Crohn’s from lupus enteritis (Table 1).

**Table 1:** The Differences between Lupus-like Crohn’s disease and Lupus Enteritis

	Lupus-like Crohn’s disease	Lupus Enteritis
Clinical Presentation	No specific (abdominal pain, diarrhea)	No specific (abdominal pain, nausea and vomiting)
Abdominal CT scan	Comb’s sign, Segmental bowel stenosis	Comb’s sign, Target sign, Pseudoobstruction, Segmental bowel dilatation
Endoscopy	Cobblestone changes, Segmental and jumping lesions	Multiple round- or oval-shaped discrete ulcers.
Therapy	5-ASA, Corticosteroids, Immunosuppressants, Biological agents	Corticosteroids, Immunosuppressants,

Steroids are considered to be first-line therapy for lupus enteritis. Depending on the clinical state or other organ involvement, steroid administration may be intravenous or oral, preferably in the case of a severe lupus burst, as tissue edema caused by enteritis may reduce drug absorption [11]. Hydroxychloroquine, azathioprine, mycophenolate mofetil could be considered for long-term maintenance treatment, although it is unclear whether recurrence can be prevented.

Here, we report a case of lupus enteritis masquerading as CD which indicated us there are some similar points between lupus enteritis and CD. Firstly, “comb’s sign” can be shown in CT of lupus enteritis or CD. Secondly, ganglion cell is not specific for the CD which can be seen in lupus enteritis. Lastly, the two diseases have similar clinical symptoms including abdominal pain and diarrhea. The endoscopic appearance of lupus enteritis is nonspecific, on the basis of our case, the features of lupus enteritis can be described as spacious, clean and no moss ulcers which discontinuous involved all gastrointestinal tract. More research and cases are needed to further identify the incidence, clinical manifestations, imaging characteristics and endoscopic appearance of lupus enteritis.

## Data Availability

The datasets used and/or analysed during the current case reports are available from the corresponding author on reasonable request.

## Abbreviations

CD: Crohn’s disease
CRP: C-reactive protein
CT: Computed tomography
ESR: Erythrocyte sedimentation rate
SLE: Systemic lupus erythematosus
